# The Transactions

**Published:** 1898-06

**Authors:** 


THE TRANSACTIONS.—TEXAS STATE MEDICAL ASSOCIATION, 1897.—Do any of the JOURNAL'S readers recall an editorial some years back, entitled "Retrench or Bankrupt," and to which Secretary West took great exceptions? Well, the retrenchment came after the bankruptcy, and at the 1897 meeting at Paris, Texas—the Association being some $200 or $300 in debt, the seventy odd members present were called upon for a "voluntary contribution," the committee on ways and means heading the list, each, with a V to pay the debt. The Secretary had been ambitious, he said, to give the Association a volume of Transacactions "worth looking at." Well, he did it. One cannot have his cake and eat it, too. The volume this year is, consequently, a very cheap affair, (not "worth looking at,") a little paper-covered pamphlet of 200 pages—(the size of two issues of the JOURNAL)—with all the discussion left out. Like Mr. Bailey's nose, (see Martin Chuzzlewit) there is none of it "worth mentioning," or, as the darkey said of the catfish, "Here you *is*; but golly, how you is swunk up."

